# MEFV gene mutations and cardiac phenotype in children with familial Mediterranean fever: a cohort study

**DOI:** 10.1186/1546-0096-12-5

**Published:** 2014-01-16

**Authors:** Samia Salah, Ranya Hegazy, Rasha Ammar, Hala Sheba, Lobna AbdelRahman

**Affiliations:** 1 Department of Pediatrics, Faculty of Medicine, Cairo University, Giza, Egypt; 2 Department of Clinical Pathology, Faculty of Medicine, Cairo University, Giza, Egypt

## Abstract

**Background:**

Familial Mediterranean fever (FMF) is the most common autoinflammatory disorder in the world. It is characterized by recurrent febrile inflammatory attacks of serosal and synovial membranes. MEFV gene mutations are responsible for the disease and its protein product, pyrin or marenostrin, plays an essential role in the regulation of the inflammatory reactions. Although the disease may carry a potential for cardiovascular disorders because of sustained inflammation during its course, the spectrum of cardiac involvement in children with FMF has not been well studied. We aimed at defining the frequency and spectrum of cardiac affection in children with FMF. The correlation between these affections and MEFV gene mutations was searched for to establish the relationship between cardiac phenotype and the patient's genotype in FMF.

**Methods:**

The present work is a cohort study including 55 patients with the clinical diagnosis of FMF based on the Tel-Hashomere criteria, confirmed by genetic analysis showing homozygous or compound heterozygous mutation of MEFV genes. Fifty age- and sex-matched normal children were included as controls. The entire study group underwent detailed cardiac examination, 12-lead ECG and echocardiography. All data was statistically analysed using SPSS version-15.

**Results:**

Patients had an average age of 8.5+/−4.2 years; with an average disease duration of 2.1+/−2.2 years; 28 were males. All controls showed no MEVF gene mutations. The most frequent gene mutation of the studied cases was E148Q mutation seen in 34% of cases and the most frequent compound mutation was E148Q/V726A seen in 16.6% of cases. Echocardiographic examination revealed pericardial effusion in nine patients. Twelve had aortic regurgitation; nine had mitral regurgitation and six had pulmonary regurgitation. The most common mutation associated with pericardial effusion was E148Q/V726A in 5/9 of cases. Valvular involvement were significantly more common in FMF patients with gene mutations. Also cardiac involvement was more common in patients with positive consanguinity. However, these cardiac manifestations showed no correlation to age, family history of FMF, or response to therapy or laboratory data.

**Conclusions:**

In our cohort of children with FMF, cardiac involvement appears to be common. Pericardial effusions are significantly related to presence of mutation types E48Q, P 369S, V726A. These associations may warrant genetic screening of children with FMF to detect cardiac risk.

## Background

Familial Mediterranean Fever (FMF) is the most common autoinflammatory. Disorder in the world [1]. It is a genetic disorder inherited as an autosomal recessive disease [2] and is characterized by recurrent febrile inflammatory attacks of serosal and synovial membranes. FMF predominantly affects the populations arising from the Mediterranean basin and seen in nearly every country [3]. Its phenotype includes recurrent attacks of peritonitis, pleuritis, pericarditis, synovitis, arthritis, and fever [4]. The Mediterranean gene (MEFV) mutations are responsible for the disease. Its protein product, pyrin or marenostrin, plays an essential role in the regulation of the inflammatory reactions. The MEFV gene contains 10 exons and most of the mutations have been found on the last exon. Up to recently, 152 mutations and polymorphisms have been reported [5].

*A*lthough the disease in adults and children may carry a potential for cardiovascular disorders because of the sustained systemic inflammation during its course [6], the spectrum of cardiac involvement in children with FMF has not been well studied [4]. Impairment of diastolic function parameters [3], pericarditis and pericardial effusion [7], atrial mechanical delay, increased *P* wave dispersion [8], and impaired coronary microvascular function [2], have all been reported in various studies. The present work aimed at defining the frequency and spectrum of cardiac manifestations in Egyptian children with FMF. The correlation between these manifestations and MEFV gene mutations was searched for to establish the relationship between cardiac phenotype and the patient’s genotype in childhood FMF.

## Methods

The present work is a cohort study that included 108 children with the clinical diagnosis of Familial Mediterranean Fever. These patients were followed at the Rheumatology Clinic of the Cairo University Specialized Pediatric Hospital. IRB approval was obtained and a written informed consents were taken from all parents.

For the purpose of this study, the diagnosis of Familial Mediterranean. Fever was based on the Tel-Hashomere criteria. Patients included fulfilled 2 major or 1 major and 2 minor criteria [9].

### Major criteria

1. Recurrent febrile episodes accompanied by peritonitis, synovitis or pleuritis.

2. Amyloidosis of AA-type without predisposing disease.

3. Favorable response to continuous colchicine treatment.

### Minor criteria

1. Recurrent febrile episodes.

2. Erysipelas-like erythema.

3. FMF in a first-degree relative.

FMF patients were included if under 16 years regardless of sex, duration of FMF, age of onset, duration of FMF attacks, frequency of attacks, family history of FMF, or clinical presentation. Fifty age and sex matched normal children were included as controls.

### Data collected

#### Detailed history

History of FMF including:

a. Patient's age at inclusion and age at onset of FMF.

b. Duration of FMF, duration & frequency of FMF attacks.

c. History of the presenting features (e.g., typical attacks of fever, abdominal pain, chest pain, joint disease, skin affection, muscle pain, scrotal pain, and history of vomiting).

d. History of renal involvement (amyloidosis, proteinuria or haematuria).

e. History of a laparotomy.

f. History of colchicine medication and response to therapy.

g. Family history including: parental consanguinity, history of FMF and history of siblings with FMF.

#### Complete clinical examination

The exam included vital signs and anthropometric measurements for height, and weight. Body mass index was calculated as weight (Kg)/height (m^2^). A detailed physical examination was performed with a particular emphasis on the cardiac examination.

#### Laboratory investigations

a. 6 ml of venous blood was collected under aseptic conditions for CBC, ESR and CRP.

b. Mutation analysis: 3 ml of blood was collected for mutation analysis.

Genomic DNA was extracted from the whole blood according to the standard procedures using QlAamp Spin Columns by QlA amp DNA Blood Kits [Cat.No.51104] [10]. The MEFV gene contains 10 exons and most of the mutations have been found on the last exon. As of this publication submission, 152 mutations and polymorphisms have been reported. Five mutations account for 70% of the deleterious alleles: M694V, V726A, M680I, M694I, E148Q. Four other mutations are less frequent: A744S, I692del, R761H, F479L. Other mutations detected were P369S, K695R. These eleven mutations were searched for. Specimens and DNA extraction: Genomic DNA was extracted from acid citrate-dextrose-anticoagulated whole-blood specimens using the Genovision GenoM −96 platform according to the manufacturer's instructions.

Detection of FMF mutations: A laboratory-developed test using the ABIPRISM SNaPshot Multiplex kit, regions of exons 2, 3, 5, and 10 of the MEFV gene were amplified by multiplex polymerase chain reaction (PCR) and the amplicons were used as templates in single base-pair extension (SNaPshot) reaction.

Mutations in the 158 samples were assessed by amplifying genomic DNA with use of primers [11]. M694V, V726A, M680I, M694I, E148Q, A744S, I692del, R761H, F479L, P369S, K695R mutations were analyzed by amplification refractory mutation system (ARMS).

#### Mutation detection by ARMS

The ARMS assay comprises two complementary reactions each conducted with the same substrate DNA. One reaction includes an ARMS primer specific for the normal DNA sequence and cannot amplify mutant DNA at a given locus. The second reaction includes a mutant- specific primer and cannot amplify normal DNA. The same common primer is used in both reactions.

The lack of polymerase chain reaction (PCR) products according to the use of a specific mutation primer set in patients suspected of carrying the mutation of FMF suggests that the patient in question is not carrying the mutation being probed [12]. However, an appropriate internal PCR control should be run to show that the DNA is amplifiable. Therefore, the complementary reaction with the normal primer set serves as an internal control for PCR amplification and allows discrimination of homozygotes from heterozygotes [12].

Mutations were assessed by amplifying the genomic DNA template with three sets of normal and mutant-specific ARMS primers designed to selectively amplify the normal or altered sequence of each of the three *MEFV* mutations. Each set of primers consisted of three oligonuleotides. Each DNA sample was tested for the twelve mutations. The PCR amplification was performed in a final volume of 25 uL containing 100 ng of purified genomic DNA, 0.04 U of Ampli Tag Gold (Perkin Elmer, Branchburg, New Jersey) and its PCR buffer (contains 15 mmol of MgCL2 per U), 0.2 mmol of deoxynucleoside 5'-triphosphate mix per L (Gibco BRL, Gautherburg, Maryland), and 1 mmol of each primer.

Amplification conditions were kept the same for all the ARMS tests, and the procedure was carried out as follows: The reaction was heated to 94°C for 9 minutes for denaturation, followed by 35 cycles with denaturation at 94°C for 10 seconds, and extension at 72°C for 30 seconds. Final extension was done for 10 minutes at 72°C.

The amplified products were separated by electrophoresis on 2% agarose gel. Ethidium bromide staining of the agarose gel was used to detect the amplified fragments [13]–[15]. Further mutation screening was performed through bidirectional automated DNA sequencing using the MEFV sequence analysis kit (Canterbury Health Laboratories) to identify the compound heterozygote under complete aseptic conditions after withdrawing 0.5 ml of EDTA blood. The DNA sequence variations were identified per reference sequence.

#### Collection of urine samples

Urine samples were collected for urine analysis to detect the presence of abnormalities especially proteinuria or hematuria.

#### Electrocardiogram (ECG)

All routine measurements were taken and any ECG abnormalities were noted.

#### Echocardiography

Echocardiographic examination was performed at the echocardiography lab CUSPH of Medicine Cairo University. Transthoracic two dimensional (2D) guided, (M. mode), color Doppler echocardiogram, and continuous wave Doppler CW were performedwith a Hewlett-Packard 5500 ultrasonic machine phased array sector scanner with the 4 and 8 probes according to age. Linear measurements of LV cavity were obtained. Left ventricle end diastolic diameter (LVEDD), left ventricle end systolic diameter (LVESD), interventricular septum (IVS) and posterior wall (PW) and calculation of fractional shortening (FS%) as an indicator of LV systolic function were done according to the recommendations of the American Society of Echocardiography. FS value < 28% were considered lower than normal with impaired LV systolic function [16]. Left ventricular mass (*LVM*) was estimated by using the anatomically validated formula of Devereux et al. [17]. Myocardial performance index (MPI), early diastolic peak flow velocity (A) and E/A ratio were calculated [18].

The echocardiographers were blinded as to whether the examined study participant was a case or a control. Data were statistically described in terms of range, mean ± standard deviation (± SD), frequencies (number of cases) and percentages when appropriate. Comparison of quantitative variables between the study groups was done using Student t test for independent samples in comparing 2 groups when normally distributed and Mann Whitney U test for independent samples when not normally distributed. For comparing categorical data, Chi square (c2) test was performed. Exact test was used instead when the expected frequency was less than 5. A probability value (p value) less than 0.05 was considered statistically significant. All statistical calculations were done using computer programs Microsoft Excel 2007 (Microsoft Corporation, NY, USA) and SPSS (Statistical Package for the Social Science; SPSS Inc., Chicago, IL, USA) version 15 for Microsoft Windows.

## Results

The demographic data of the study group is shown in Table 1. Abdominal pain was the most common clinical presentation of FMF in our group (n = 106, 98%) followed by fever in 102 (94.4%) patient. Other manifestations are shown in Table 2. The laboratory investigations of the FMF cases showed that 53.7% (n = 58) had positive urinary casts, 31.5% (n = 34) had oxalates and 20.4% (n = 22) had proteins in their urinanalysis. Twelve percent (n = 13) had high CRP levels. Erythrocyte sedimentation rate, creatinine levels and CBC are shown in Table 3. All controls showed no MEVF gene mutation. Five (4.6%) of the FMF cases showed normal gene analysis while 103 (95.4%) showed gene mutations. Two female FMF cases showed homozygosity while the rest were heterozygous. Fifth-three (49%) showed compound heterozygous mutations. The different gene mutations are shown in Tables 4 and 5.

**Table 1:** Demographic data of the clinically diagnosed FMF

	**Cases (n = 108)**	**Controls (n = 50)**	**P value**
**Mean age**	8.5 ± 4.2	8.6 ± 3.9	0.78
**Range**	2–17	3-15	
**Sex**			
**Male**	55(50.9%)	23(46%)	0.47
**BMI**	21.2 ± 5.1	24.5 ± 6.7	0.69
**+ve FH of FMF**	21(19.4%)	0	0.02
**+ve consanguinity**	43.(39.8%)	20(10%)	0.21
**Age at onset**	6.3 ± 11.3 ys	-	
**Time to diagnosis**	1.2 ± 0.4 ys		
**Duration**	2.1 ± 2.2 ys		

BMI: body mass index FH: Family history.

**Table 2:** Clinical Manifestations of the study group

**Clinical manifestations**	**No**	**Percent**
**Abdominal pain**	106	98.1%
**Fever**	102	94.4%
**Arthralgia**	84	77.8%
**Myalgia**	81	55.0%
**Arthritis**	54	50.0%
**GIT$**	53	49.1%
**Chest pain**	47	43.5%
**Skin rash**	41	38.0%
**Neurological symptoms**	11	10.2%
**Appendicectomy**	3	2.7%
**Scrotal involvement**	2	3.6%
**Renal symptoms**	0	0.0%

GITS: gastrointestinal symptoms.

**Table 3:** Laboratory Investigations of the Study Group

	**Mean**	**SD**	**Median**	**Range**
**ESR mmHg**	30.9	31	19	5–150
**Creatinine mg/dl**	0.53	0.13	0.5	0.1–1
**HB g/dl**	11.1	1.5	11.3	6.2–14
**TLC**	8.1	4.4	7.2	3–31
**Neut. %**	51.1	16.7	50	17–86

ESR: erythrocyte sedimentation rate. TLC: total leucocytic count.

**Table 4:** The different mutation types of MEFV gene among the cases

			**Cases**
**Mutation type**	**E148Q**	Count	35
		% within group	34.0%
	**M680I**	Count	19
		% within group	18.4%
	**M694I**	Count	14
		% within group	13.6%
	**P369S**	Count	6
		% within group	5.8%
	**V726A**	Count	29
		% within group	28.2%
**Total**		Count	103
		% within group	95.4%

**Table 5:** Compound mutations among the FMF cases

			**Cases**
**Mutation type**	**E148Q/M680I**	Count	10
		% within group	34.0%
	**E148Q multiple allele**	Count	7
		% within group	6.4%
	**E148Q/M6941**	Count	6
		% within group	18.4%
	**E148Q/V726A**	Count	18
		% within group	13.6%
	**V726A multiple allele**	Count	4
		% within group	3.7%
	**M680I/P369S**	Count	3
		% within group	5.8%
	**M6941/V726A**	Count	5
		% within group	28.2%
**Total**		Count	53
		% within group	49%

The most frequent gene mutation of the studied cases was E148Q mutation, seen in 34% of cases and the most frequent compound mutation was E148Q/V726A (16.6%). Importantly, all patients with simple heterozygous mutations were excluded from the present work.

Seventy-four percent (n = 39) of the FMF patients showed a good response to therapy. This “good response” was defined by less than 2 attacks/year while 26% (n = 14) showed fair response, defined by 4–5 attacks/year. None of our FMF patients were resistant to treatment.

### Cardiac evaluation of FMF patients

Thirty-nine FMF cases (74%) had cardiac complaints; the most common of which was palpitations in 59% (Table 6). Many patients showed more than one complaint. All patients had normal blood pressure. Echocardiographic testing revealed pericardial effusions in 9 patients. Seven had mild and 2 had moderate effusions. None had cardiac tamponade. Twelve had aortic regurgitation: 8 had grade I and 3 had grade II. Nine had mitral regurgitation: 5 had grade I, 3 had grade II, and 1 had grade III. Six had pulmonary regurgitation; all 6 were grade I. All patients had sinus rhythm with normal axis. No significant differences were noted on 12 lead ECG between both the two groups.

**Table 6:** Cardiac manifestations seen in 39/55 cases

		**Group cases (n = 55)**
**Dyspnea**	No	17
	%	31%
**Palpitation**	no	23
	%	59%
**LL oedema**	no	4
	%	7.2%
**Syncope**	no	5
	%	9%

### Relation between cardiac involvement and gene mutations

The most common mutation associated with pericardial effusion was E148Q/V726A (5/9 cases). The next most common association was the E148Q multiple allele mutation (3/9 cases). The M680I/ P369S mutation was present in 1/9 of cases. Valvular involvement of the heart was significantly more common in FMF patients with gene mutations and only one patient with no FMF gene mutation had mild mitral regurgitation. Cardiac involvement was significantly more common in patients with compound heterozygous mutations (n = 27) as compared to those with simple mutations (n = 12, p = 0.003). The distribution of valvular disease among the different gene mutations are shown in Table 7. E148Q mutation was the most commonly identified mutation among FMF patients with cardiac involvement. Cardiac involvements were more common in patients with positive consanguinity (p = 0.035). However, cardiac disease in these patients showed no correlation to age, family history of FMF, response to therapy, or laboratory data.

**Table 7:** Frequency of valvular lesions among the different Gene mutation type

		**Mutation type**						
		**E148Q/M680I**	**E148Q**	**E148Q/M6941**	**E148Q/V726A**	**V726A**	**M680I/P369S**	**M6941/V726A**
**Pulmonary (n = 6)**	no	1	1	2	0	2	0	0
	%	16.6%	16.6%	33.3%	0.0%	33.3%	0%	0%
**Mitral (n = 9)**	no	2	3	0	1	3	0	0
	%	22.2%	33.3%	0.0%	11.1%	33.3%	0%	0%
**Aortic (n = 12)**	no	4	2	4	1	0	0	1
	%	33.3%	16.6%	33.3%	8.3%	0%	0%	8.3%

## Discussion

Defining children with FMF has proved more challenging than previously thought. FMF has traditionally been considered an autosomal recessive disease. However clinical phenotypes among patients who are heterozygous for FMF has been repeatedly reported and the genetics of FMF has been found more complex than previously appreciated, suggesting partial penetrance and variable expression [19].

Heterozygous FMF patients tend to have mild disease. Patients with other inflammatory conditions as Behcet’s disease and tuberculosis [20] have been found to test positive for MEFV gene mutation. The vast majority of these Behcet’s and TB patients have single mutations [21]. For patient selection we have excluded heterozygous FMF patients with single gene mutations and included only homozygous FMF patients and those with compound heterozygous mutations.

The present work reports an incidence of pericarditis of 16.4% and valvular involvement of 49% in these children with these homozygous and compound heterozygous FMF mutations. The valvular disease detected consisted of aortic (21.8%), mitral (16%), and pulmonary regurgitation (11%) involvement.

In a study by Maisch et al. [22], 5% of patients with FMF (adults) were found to present to the emergency department for non-acute myocardial infarction-type chest pain mainly accounted for by pericarditis. Recurrent pericarditis may occur in 15-30% of FMF patients. Examination of the pericardial fluid typically reveals activation of proinflammatory cytokines such as IL-6, IL-8, and interferon gamma [23]. Reports on the frequency of pericarditis in FMF are quite variable. In a retrospective study by Kees et al. [24] that included 4000 FMF patients during a 20 year period, the prevalence of pericarditis was reported to be 0.7%. The Turkish FMF study group reported the incidence of pericarditis to be 1.4% in a cohort study [25]. Dabestani etal. [26], though, reported a much higher prevalence (27%) of pericarditis in patients with FMF. The often asymptomatic course of pericarditis in most cases of FMF and the various diagnostic methods of detecting pericarditis are most likely responsible for these variations [27].

Certain mutations in the MEVF gene result in uncontrolled neutrophil activation and inflammation in several tissues. However, the present work suggests that the mutations in the E 148Q and V726A are most commonly associated with pericarditis in contrast to other mutations. This finding suggests that cardiac screening in patients with FMF gene mutation prone to pericarditis may be useful. It is important to emphasize for the clinician that FMF should be also considered in children presenting with non-infectious pericarditis. Cardiac functions were well preserved in our patients with FMF.

Previous studies have shown that left ventricular diastolic function indices are commonly impaired in FMF patients [28]. Systolic dysfunction and cardiac failure commonly follow these alterations in LV diastolic functions [29]. None of these changes were observed in our patients. This may be due to the younger age of our study population and hence the shorter duration of disease and less time for chronic changes, as it appears that the main pathophysiologic mechanism for these impairments may be the chronic inflammation over time. This chronic inflammation through a cumulative effect may accelerate the development of atherogenesis and thrombosis [28]. Subclinical amyloidosis might be another factor in the pathogenesis of diastolic dysfunction in this disease, again a pathology progressive with time [30].

On the other hand, cardiac function has been investigated in children with FMF by using conventional & tissue Doppler imaging [3]. Systolic function was found to be normal, while diastolic dysfunction was reported. This was not observed in this study. Valvular involvement was noted in 27 (49%) of our patients. No correlation was found between valvular involvement & the type of genetic mutations. However, none of the children with FMF showed valvular disease in the absence of genetic mutations. Further studies including a larger number of children will be needed to further evaluate these associations.

## Conclusions

Cardiac involvement is common in children with FMF. Pericardial effusions are significantly related to the presence of mutation types E 148Q, P 369S, V726A. These associations suggest the possibility that genetic screening of FMF patients to detect risk for significant FMF heart involvement may be prudent.

## Abbreviations

FMF: Familial Mediterranean fever; MEFV: Mediterranean fever gene; PCR: Polymerase chain reaction.

## Competing interest

The authors declare that they have no competing interest.

## Authors’ contributions

SS carried out study design, analysis of data and revision of manuscript. RH participated in acquisition and analysis of data, carried out drafting and submitting the manuscript. RA participated in study design, acquisition and analysis of data. HS participated in acquisition and analysis of data. LM participated in study design, acquisition and analysis of data. All authors read and approved the final manuscript.
